# Rethinking Extracellular Vesicle Signaling

**DOI:** 10.1002/adma.202522172

**Published:** 2026-03-11

**Authors:** Wojciech Chrzanowski, Joy Wolfram

**Affiliations:** ^1^ Sydney Pharmacy School Faculty of Medicine and Health The University of Sydney Camperdown New South Wales Australia; ^2^ Division of Biomedical Engineering Department of Materials Science and Engineering Uppsala University Uppsala Sweden; ^3^ School of Chemical Engineering The University of Queensland Brisbane Queensland Australia; ^4^ Australian Institute for Bioengineering and Nanotechnology The University of Queensland Brisbane Queensland Australia

**Keywords:** bind‐and‐leave, bind‐and‐stay, ectosome, exosome, internalization, microvesicle

## Abstract

Extracellular vesicles mediate intercellular communication through transport of bioactive cargo from producing to recipient cells. Cellular uptake and subsequent cytoplasmic release of internal cargo is often emphasized as the main mechanism of extracellular vesicle‐mediated signaling. This article highlights two alternate modes of extracellular vesicle signaling based on surface binding without internalization: ‘bind‐and‐stay’, and ‘bind‐and‐leave’. The transient binding of extracellular vesicles to multiple cells, inducing downstream signaling in each, challenges the conventional “one‐vesicle‐one‐cell” model. We provide a forward‐looking perspective on the efficiency and effectiveness of extracellular vesicle signaling modes and outline techniques that could be leveraged to study these interactions moving forward. An improved understanding of extracellular vesicle signaling is necessary for advancing fundamental biology and therapeutic development.

## Graphical abstract

Extracellular vesicles enable cell communication beyond intracellular cargo delivery. This perspective highlights two plausible surface‐based signaling modes: “bind‐and‐stay” and “bind‐and‐leave.” Transient binding to multiple cells challenges the one‐vesicle‐one‐cell model. We discuss implications for signaling efficiency and outline methods to study these mechanisms, advancing biological understanding and therapeutic development.

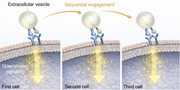

## 1 Introduction

Extracellular vesicles (EVs) are cell‐produced biomolecular packages delineated by lipid bilayers. Historically, EVs were primarily considered waste disposal units for obsolete biomolecules [1]. Over the past decades, EVs were shown to also mediate intercellular communication between producing and recipient cells, including modulation of messenger RNA (mRNA) and microRNA (miRNA) levels in target cells, thereby, affecting gene regulation and various biological processes, including cell migration, differentiation, cellular homeostasis and mineralization [2]. The role of EVs in intercellular communication makes them promising as mechanistic, therapeutic, and diagnostic agents in health and disease [3, 4]. However, the relative contribution of various modes of EV signaling remains largely unknown, which impedes progress in the field. Additionally, assuming that certain signaling pathways are dominant can result in misleading conclusions.

**FIGURE 1:**
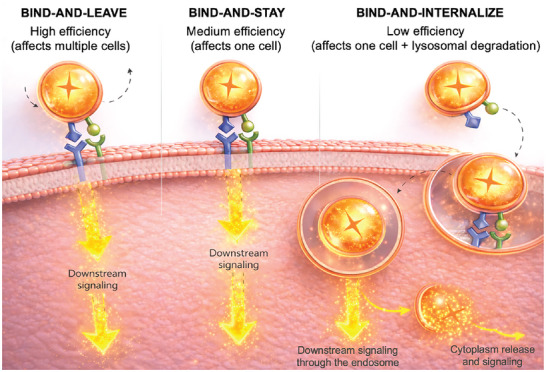
Plausible modes of extracellular vesicle (EV) signaling. The ‘bind‐and‐leave’ mode involves temporary binding of EVs to the recipient cell surface, eliciting downstream signaling. The ‘bind‐and‐leave’ mechanism enables one EV to affect multiple cells through transient interactions, leading to high efficiency. An example of the bind‐and‐leave mechanism is programmed death‐ligand 1 (PD‐L1) on EVs transiently binding to programmed cell death protein 1 (PD‐1) on cells. The ‘bind‐and‐stay’ mode involves prolonged binding of EVs to the recipient cell membrane without internalization. An example of the bind‐and‐stay mechanism is antigen‐containing EVs attached to the surface of dendritic cells. The ‘bind‐and‐internalize’ mechanism has low efficiency in terms of impacted cells, as the EV usually stays within the internalized cell and endosomal escape levels are low. It is worth noting that internalization may still be an effective mode of signaling, especially if protein and RNA cargo have prolonged and multipronged effects through transcriptional and translational regulation.

This article highlights three plausible modes of EV signaling with varying degrees of effectiveness and efficiency: ‘bind‐and‐leave’, ‘bind‐and‐stay’, and ‘bind‐and‐internalize’ (Figure 1). Generally, efficiency means optimal use of resources, while effectiveness is achieving objectives. In the context of EVs, effectiveness represents inducing a response in a target cell, while efficiency is the speed and quantity of successful interactions. Efficiency can be assessed through parameters, such as, EV binding rates, persistence of EVs, and cargo release kinetics. Conversely, effectiveness reflects the extent to which EVs achieve their functional outcomes, namely, a change in target cells that produce a measurable biological effect. Effectiveness can be evaluated through target gene expression, phenotypic changes in recipient cells, or functional assays.

The heterogeneity and nanoscale dimensions of EVs make it technically challenging to probe signaling, leaving many important questions unanswered. Recognizing and considering diverse modes of EV signaling opens new avenues for studying and refining mechanistic models. This broader perspective also drives the development of innovative tools, ultimately advancing our fundamental understanding of EV biology. This article discusses a range of techniques that offer opportunities to identify the relative contributions of cell surface signaling versus uptake on EV‐mediated effects.

In addition to advances in basic knowledge, an increased understanding of EV signaling is important for the development of EV‐based therapeutics. Several EV therapeutics have already progressed to late stage clinical trials [5], and signaling modes are likely to influence both safety and efficacy. Therapeutic EVs can also be chemically or genetically engineered to further promote signaling effects [6, 7]. Identifying the dominant signaling pathway underlying the mechanism of action is essential for tailoring engineering strategies that amplify therapeutic effects. For example, therapeutic mechanisms that are dependent on the cytoplasmic release of EV cargo in recipient cells may benefit from engineering strategies that reduce lysosomal destruction. Conversely, EV therapeutics that rely on surface signaling may benefit from engineering strategies that prevent cellular internalization. Additionally, understanding EV signaling modes is critical for developing therapeutic strategies to block pathological EVs that drive disease.

This article explores outstanding questions about the modes, efficiency, and effectiveness of EV signaling and their implications for basic biology and therapeutic development.

## 2 EV Signaling Modes

### 2.1 Cellular Uptake of EVs

**FIGURE 2:**
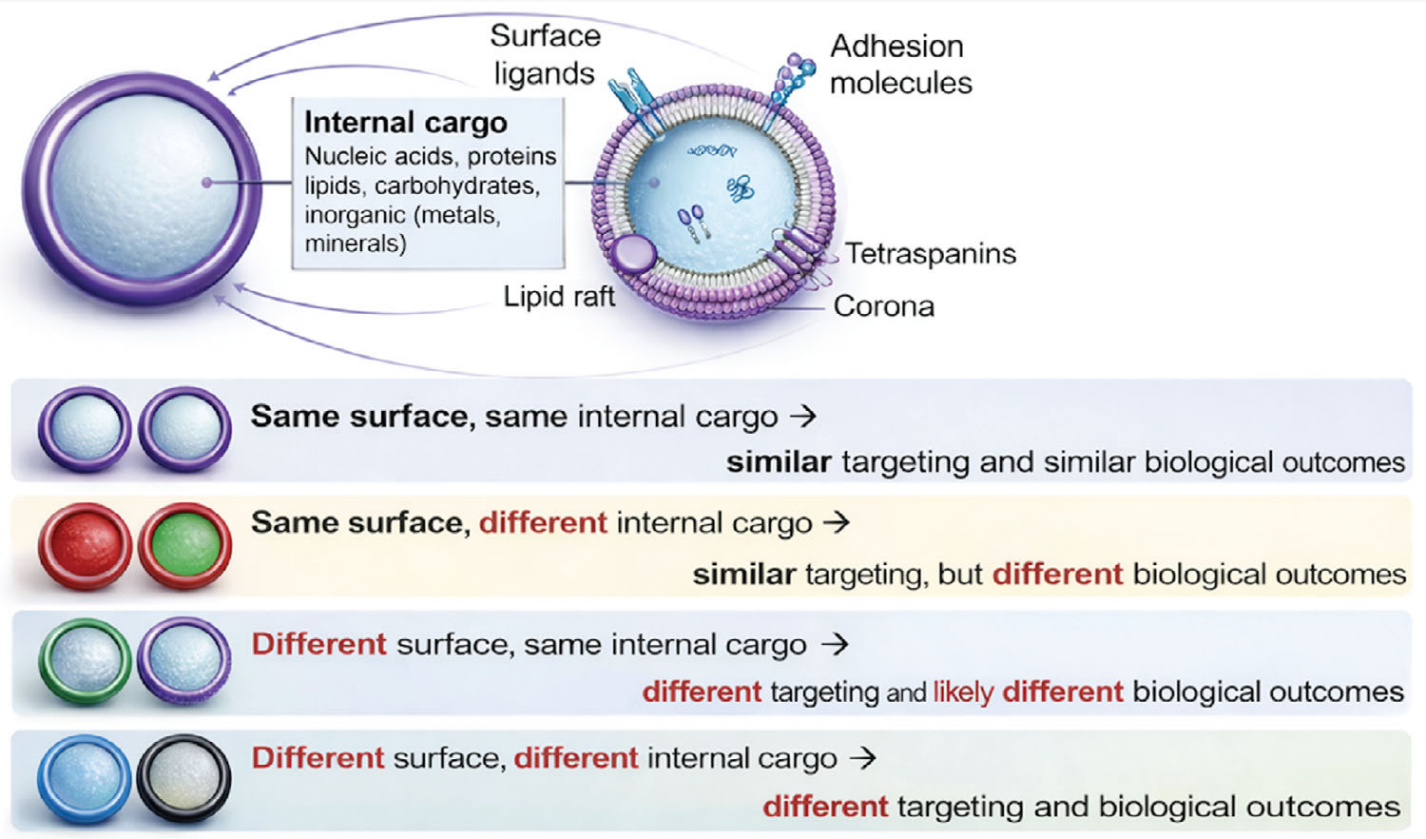
Impact of EV heterogeneity on cell targeting/uptake and effects in recipient cells. Four possible scenarios of varied EV surface and internal cargo composition that impact EV signaling. Each color represents a distinct internal cargo or surface composition.

EV‐mediated communication is often portrayed as occurring through internalization and cytoplasmic release of EV cargo in target cells. Endocytosis is the predominant mechanism of EV uptake in cells, although complete or transient fusion of EVs with the recipient cell membrane may occur in rare cases [8, 9]. Endocytosis of EVs can occur through clathrin‐dependent endocytosis, caveolae‐dependent endocytosis, clathrin/caveolae‐independent endocytosis, micropinocytosis, and phagocytosis [8, 9, 10]. EV binding to target cells, pathways of uptake, and subsequent effects in recipient cells depend on both surface composition and internal cargo, leading to four overarching scenarios: i) identical surface and internal cargo results in the same targeting and biological effects, ii) identical surface but distinct internal cargo enables targeting the same cells with potentially different biological outcomes, iii) distinct surface composition but similar internal cargo targets different cells, and likely induces different biological effects, and iv) distinct surface and internal cargo directs EVs to different targets and induces varied biological effects (Figure 2).

In general, internalization is an inefficient mode of signaling, as one EV impacts a single recipient cell. Additionally, only a small portion of internalized EV cargo escapes the endosome [8, 11, 12], limiting the extent of signaling that can occur. EV‐mediated endosomal escape mechanisms remain largely unknown and endosomal escape is dependent on the originating and recipient cell type. Studies showed that identical EVs are processed in substantially different ways depending on the recipient cell. For example, the same EVs displayed limited lysosomal escape in tissue‐resident macrophages, while endosomal escape levels were much higher in hepatocytes [13].

The exception to the one‐to‐one ratio between internalized EVs and recipient cells is the re‐release of EVs [8]. It is possible that the surface cargo of EVs could affect signaling in the endosome, prior to being re‐released, thereby, affecting multiple cells despite initial internalization. However, in the case of re‐release of intact EVs, the internal content is unlikely to affect the initial recipient cell [8]. It might be possible for EVs to partially release their cargo through temporarily opening the phospholipid bilayer or shedding lipid bilayers, in the case of multilayered EVs [14], prior to being re‐released.

It is worth emphasizing that the low efficiency of internalization‐mediated EV signaling does not equate to low effectiveness. EV internalization remains a potentially effective mode of signaling, as cytoplasmic delivery of protein and RNA cargo can have prolonged and multipronged effects through transcriptional and translational regulation [15, 16, 17, 18, 19, 20, 21, 22, 23, 24]. There are numerous instances where inefficient biological processes lead to effective outputs, for example, more than 90%–95% of developing T cells [25] and B cells [26], are eliminated, yet the adaptive immune system is highly robust.

### 2.2 EV Surface Signaling

The relative contributions of surface signaling vs. internalization to EV‐mediated effects on recipient cells have been overlooked, and uptake is often assumed to be the primary mechanism. Compared to cells, the surface area to volume ratio is much larger for EVs (> 100‐fold), making them ideally suited for surface signaling vs. signaling through internal cargo. There are also several advantages of EV surface signaling compared to signaling through soluble biomolecules. For example, EVs can enable protection, clustering, integration with co‐signals, and altered biodistribution of surface biomolecules [27, 28]. Notably, a study demonstrated that the signaling potential of ligands increases by over 100‐fold when immobilized in EV‐like vesicles compared to being in soluble form [28]. Additionally, hydrophobic biomolecules require a lipophilic environment for effective extracellular transport and signaling with recipient cells. Signaling through wingless‐related integration site (Wnt) proteins, which are lipophilic, can take place through their incorporation in the exterior surface of EVs [29]. In addition to hydrophobic molecules, EV‐surface signaling also occurs with biomolecules known to have soluble forms, including cytokines [30] and TNF‐related apoptosis‐inducing ligand (TRAIL) [31], the latter of which conveys proapoptotic signals by binding to TRAIL‐death receptor 5 on cancer cells. Other examples include cancer cell‐derived EVs that suppress T cells through surface intercellular adhesion molecule‐1 (ICAM‐1), which binds to lymphocyte function‐associated antigen‐1 (LFA‐1) on the cell surface [32]. Additionally, EVs from cancer cells can also contain transmembrane immune checkpoint proteins, such as programmed death‐ligand 1 (PD‐L1), which binds to programmed cell death protein 1 (PD‐1) to suppress T cells [33]. It is important to note that in addition to proteins, other biomolecules, such as glycans [34] and lipids [35] also partake in EV surface signaling. For a comprehensive overview of EV surface signaling readers are referred to articles by Buzas et al. [36]. and Hallal et al. [30].

The spatiotemporal distribution of ligands is a key parameter that is likely to modulate EV interactions with cells and subsequent biological processes. Studies demonstrated that the density, mobility, and clustering of cell‐surface ligands substantially impacted binding affinities and subsequent cellular responses [37]. The EV surface could act as a scaffold that brings together several ligands, each with distinct receptor pairs on the cell surface [27]. Some ligands may primarily be involved in adhering EVs to cell surfaces, while others trigger downstream signaling. A study demonstrated that cluster of differentiation (CD)9 plays an important role in adhering vesicles to cell membranes, which likely facilitates downstream signaling induced by ligand‐receptor binding, for example, LFA‐1 to ICAM‐1 [38]. Although the ratios of various CDs differ substantially based on the size and origin of EVs [39], the potential impact of this ratio on surface signaling remains unknown. Multiple interactions among participating EV surface molecules are likely to enable fine‐tuning and nuanced control to diversify EV signaling outcomes. Signaling could be further fine‐tuned by the concurrent binding of multiple EVs to a single cell, resulting in the amplification or antagonism of downstream pathways.

It is also likely that EV surface ligands have additive or synergistic effects through horizontal proximity, resulting in more potent downstream signaling in recipient cells compared to soluble ligands [28, 40]. Studies using model nanoparticles decorated with various ligands demonstrated the existence of threshold ligand density. Beyond this threshold, further increases in ligand density did not correlate with enhanced binding or modulation of cellular functions, likely due to steric hindrance or receptor saturation [41]. For example, for folate ligands (molecular weight of 414 Da), the threshold density fell between 0.5 and 2.0 ligands per 100 nm^2^, which agreed well with the spike density of most viruses [41]. In contrast, molecules with larger molecular weight, for example, transferrin (80 kDa) and antibodies (150 kDa), plateaued at around 0.7 and 0.3 ligands per 100 nm^2^, likely due to steric hindrance [41]. In the context of EVs, comprehensive studies on ligand densities are lacking.

A more efficient form of intercellular communication compared to cellular uptake of EVs would be surface signaling through a ‘bind‐and‐leave’ mechanism where one EV impacts numerous cells through temporary binding to the surface to elicit downstream signaling. While plausible, this type of sequential signaling has not yet been fully demonstrated experimentally due to technical challenges. The ‘bind‐and‐leave’ type of EV signaling has previously been referred to as ‘kiss‐and‐run’ [40], however, this terminology can cause confusion, as it is used in neuroscience to describe partial exocytosis of synaptic vesicles. Associations between ligands and receptors are driven by reversible intermolecular (weak) forces, such as ionic and hydrophobic interactions. The reversibility of ligand‐receptor interactions mitigates receptor desensitization during prolonged exposure [42, 43]. The residence time of EVs in bind‐and‐leave type interactions would likely be milliseconds to minutes; similar to that of soluble ligand‐receptor interactions [44] and transient cell‐cell interactions [45]. The potential superiority of EV‐mediated ‘bind‐and‐leave’ signaling compared to internalization has been overlooked. Although evidence of EV cell surface binding kinetics linked to multi‐cell effector function is lacking, it would be unusual if the EV surface failed to evolve an ability to consecutively impact recipient cells.

In addition to the ‘bind‐and‐leave’ mechanism, EVs can be attached to the recipient cell membrane for prolonged periods, a mechanism that we have coined ‘bind‐and‐stay’. For example, antigen‐containing EVs can attach to the surface of dendritic cells to aid in signaling to the adaptive immune system [46]. The duration that EVs persist on the cell surface is likely to be context‐dependent, varying with EV type, cell type, and microenvironment. To enable ‘bind‐and‐leave’ and ‘bind‐and‐stay’ modes of signaling, EVs may contain surface molecules that prevent internalization. One such example is EV‐associated CD47 that binds to signal regulatory protein alpha (SIPRα) on phagocytes, triggering downstream signaling that suppresses the cytoskeleton machinery required for internalization in these cell types [47].

## 3 EVs in Circulation

**FIGURE 3:**
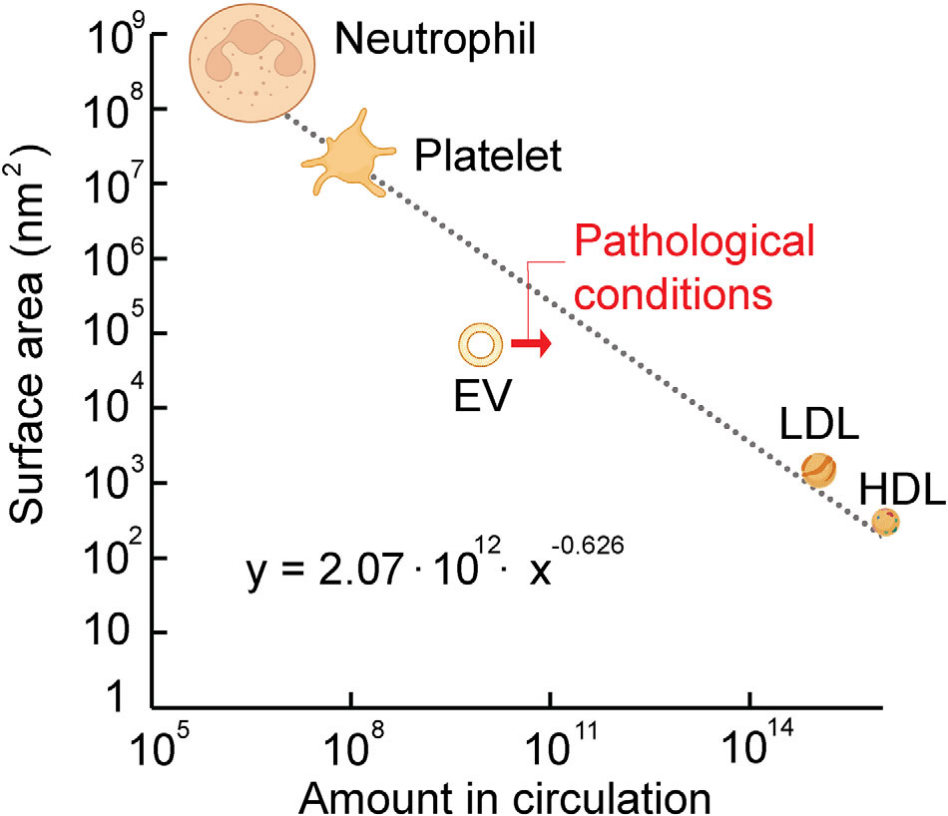
Abundance of circulating factors that display surface signaling plotted against the surface area of such factors in physiological conditions. An increased surface area is associated with decreased abundance of such factors in circulation, consistent with approximate power‐law scaling. EVs deviate the most from the fitted trend. The arrow indicates an increase in circulating EV levels commonly observed in pathological states. HDL, high‐density lipoprotein; LDL, low‐density lipoprotein.

EV signaling over long distances relies on transport through the lymphatic and/or blood circulations [10, 48]. The number of circulating EVs in plasma is predicted to be 10^10^/mL in physiological conditions [49]. However, it is challenging to determine the amount of EVs in circulation, as most isolation methods fail to completely separate EVs from contaminants [50, 51]. A particularly challenging contaminant is lipoproteins, which overlap in size and can form complexes with EVs [52, 53, 54]. Notably, circulating EVs are several orders of magnitude less abundant than lipoproteins and several orders of magnitude more abundant than platelets (Table 1). The abundance of circulating factors that display surface signaling can be plotted against the surface area. Specifically, the surface area decreases as the abundance in circulation increases, consistent with approximate power‐law scaling (Figure 3). Among various circulating components that take part in surface interactions, including neutrophils, platelets, low‐density lipoprotein (LDL), and high‐density lipoprotein (HDL), EVs deviate the most from the fitted trend (Figure 3). At present, it remains unclear whether this deviation reflects an underestimation of the EV surface area or circulation concentration, involvement of signaling mechanisms beyond surface‐mediated interactions, or none of these possibilities. A speculative interpretation is that EVs employ both surface‐mediated and internalization‐dependent signaling, setting them apart from neutrophils, platelets, and lipoproteins, which predominantly interact with cells without uptake. Notably, in pathological conditions such as cancer and inflammation, circulating EV levels usually increase [55, 56, 57, 58], reducing the deviation from the fitted trend (Figure 3) and raising the question of whether specific EV signaling modes predominate under physiological conditions compared with disease states. A disease‐associated increase in the size of circulating EVs would further decrease this deviation, however, in some cases, enhanced levels of circulating EV are accompanied by a reduction in EV size [58].

**TABLE 1:** Examples of circulating factors that engage in surface signaling.

Signaling entity	Corresponding number in 1 mL	Signaling mechanism	Refs.
HDL	10^16^	Primariy bind‐and‐leave (bind‐and‐internalize can also occur). Lipoproteins bind to various receptors on the cell surface, triggering exchange of lipids/lipophilic molecules, downstream signaling, and/or internalization.	[49, 61, 62]
LDL	10^15^	Primarily bind‐and‐leave (bind‐and‐internalize can also occur). Lipoproteins bind to various receptors on the cell surface, triggering exchange of lipids/lipophilic molecules, downstream signaling, and/or internalization.	[49, 61, 62]
EVs	10^10^	Bind‐and‐leave, bind‐and‐stay, bind‐and‐internalize.	[49]
Platelets	10^8^	Bind‐and‐leave or bind‐and‐stay. Upon activation, platelets bind to various receptors, triggering aggregation.	[63, 64]
Neutrophils	4.5 × 10^6^	Bind‐and‐leave or bind‐and‐stay. Neutrophils can bind to various receptors on the endothelial cell surface, triggering rolling.	[65, 66]

It is also important to note that EVs in circulation are highly heterogenous, as they are produced by different cells [59] and each cell type releases EV subpopulations with differing content [60]. Therefore, specific signaling ligands would be present on a small percentage of bulk EVs in circulation.

## 4 Therapeutic Implications of EV Signaling

EV signaling is leveraged for therapeutic applications, with several EV therapeutics in early and late‐stage clinical trials [5, 67]. In particular, EVs from mesenchymal stem cells suppress inflammation and promote tissue repair, with effects attributed to internal cargo, such as microRNAs (miRNA) [12]. However, studies suggest that too few miRNA molecules are present for EVs to have an effect in recipient cells [68, 69]. Several studies have increased miRNA levels in EVs through genetic engineering to obtain therapeutic responses [70, 71, 72, 73]. Taken together, these findings suggest that the therapeutic effects of native mesenchymal stem cell EVs are unlikely to be primarily driven by cellular internalization of miRNAs. The view that internal cargo mediates therapeutic effects has also been challenged by emerging evidence pointing to alternative mechanisms, such as surface signaling and modulation of the microenvironment [40, 68, 69, 74]. Uncertainties around therapeutic mechanisms of action are likely to affect the development of mesenchymal stem cell EVs in terms of efficacy, safety, and regulatory approval.

Another example of therapeutic EVs is antigen‐carrying ones for cancer immunotherapy [75, 76]. Notably, dendritic cell‐derived EVs carrying tumor antigens were the first human EVs to enter clinical trials [77, 78]. In the case of dendritic cell EVs, immunotherapeutic mechanisms may occur through cell internalization and/or cell surface interactions [46, 79].

When therapeutic effects are mediated by internal cargo release, engineering EVs to overcome the barrier of endosomal escape becomes a key consideration. Such engineering approaches include the incorporation of fusogens into EV membranes to improve fusion with cell or endosomal membranes. For example, a study showed that the fusogenic viral component, vesicular stomatitis virus G (VSV‐G) protein, enhances endosomal escape of EV cargo, enabling efficient payload delivery to the cytoplasm of target cells [80]. Incorporation of fusogens can be achieved using two primary strategies: i) genetic alterations of producing cells to express fusogenic proteins that are recombinantly attached to EV‐enriched membrane anchoring proteins and ii) chemical and physical methods to alter the surface post‐EV release (for a comprehensive review on surface modifications, see [7]). It is important to note that viral fusogens may elicit immune responses [81, 82] that accelerate the clearance of therapeutic EVs [83]. Additionally, EV engineering strategies that promote cytoplasmic delivery may induce toxicity, especially if the mechanism is based on lysosomal rupture [84] rather than back‐fusion.

When EV surface signaling plays a central role in therapeutic effects, engineering strategies that decrease cellular uptake may be preferred. If phagocytes are the target cells for surface signaling, strategies may include incorporation of ‘don't eat me’ surface proteins, such as CD47^47^ and CD24 [85], which reduce uptake in such cells. It is also important to consider the effects of the EV biomolecular corona when surface signaling is the predominant mechanism of action. The corona is a dynamic biomolecular coating that forms around EVs and cells in biological fluids [86, 87, 88, 89, 90, 91, 92]. The composition of loosely associated (soft) and tightly associated (hard) coronas are highly dependent on the local microenvironment, that is the biomolecular composition of the surrounding fluid [93, 94]. Two potential scenarios associated with corona formation include: (i) biomolecules adsorbed on the EV surface enhance binding to and signaling with cells, and (ii) coronas render EV surface molecules ‘invisible’ to cells, thereby, reducing signaling capabilities. Accordingly, it was shown that the corona can be responsible for EV signaling and subsequent therapeutic effects [87, 95]. On the contrary, studies in the field of nanomedicine demonstrated that the biomolecular corona can mask molecules on the surface of nanoparticles, which hinders binding to target cells [96], suggesting that this may also be the case for surface‐engineered EVs. Therefore, it may be preferrable to engineer EVs to attract a favorable biomolecular corona rather than directly incorporate ligands on the EV surface. Engineering strategies to attract a favorable corona have been adapted in the field of nanomedicine [97, 98, 99, 100], for example, apolipoprotein binding to cross the blood‐brain barrier [100].

An alternative strategy to attracting a favorable corona is preventing the formation of an unfavorable one. Synthetic polymers, such as polyethylene glycol (PEG), were incorporated in EVs to reduce corona opsonins, which accelerate the immunological clearance of EVs [83]. Such polymers reduce EV interactions with phagocytic immune cells, primarily those in the liver, consequently prolonging circulation time and engagement with target cells [101, 102, 103]. However, upon repeated administration, an antibody‐mediated response to PEG may occur, paradoxically resulting in rapid clearance of EVs [104]. It is also important to note that PEG can mask surface ligands, preventing receptor engagement. In the case of synthetic nanoparticles, the trade‐off between reduced clearance and exposed surface ligands was mitigated by optimizing PEG density and chain length or by using stimuli‐responsive PEG‐shedding approaches [105, 106, 107]. Cleavable PEG strategies are also beginning to be explored in the context of engineered EVs [108, 109]. In such contexts, cleavage is triggered by factors in the pathological environment, such as reactive oxygen species or low pH. Once PEG is cleaved in the pathological tissue interstitium, EVs may engage locally in bind‐and‐leave or bind‐and‐stay/internalization signaling.

In parallel with efforts to harness beneficial EVs, therapeutic strategies are emerging that block pathological EV signaling in disease, primarily in the context of cancer. Various studies have shown that cancer‐derived EVs mediate immunosuppression, tumor growth, metastasis, and chemoresistance [110, 111, 112], making them attractive as therapeutic targets. A mouse study demonstrated that a therapeutic peptide could lyse the membranes of cancer cell‐derived EVs expressing the immunosuppressive ligand, PD‐L1, thereby, reducing suppressive surface signaling with T cells and inhibiting tumor progression [113]. Similarly, a clinical study demonstrated that cancer PD‐L1‐containing EVs could be removed from circulation through therapeutic plasma exchange [114]. Additionally, a mouse study showed that administering antibodies against EV‐associated tetraspanins tagged cancer EVs for immunological destruction, consequently reducing signaling leading to metastatic progression [115]. Several preclinical anti‐cancer strategies have also been developed to block the uptake of EVs by recipient cells, including endocytosis inhibitors [116] and antibody Fab fragment against CD9 [117]. A clear understanding of EV signaling modes in disease will be critical for the development of effective strategies to block pathological EVs.

## 5 Techniques to Assess EV‐Cell Surface Interactions

Studies often fail to assess the relative contributions of surface signaling vs. internalization on EV‐mediated effects in recipient cells. Immediate surface‐dependent readouts, such as rapid phosphorylation events, calcium flux, and recruitment of surface‐bound ligands to signaling hubs, would be suggestive of EV‐mediated functional effects through surface signaling. A challenge in the evaluation of surface signaling is damage that can occur to EVs during isolation and storage. For example, ultracentrifugation can damage EV membranes [118], leading to reduced functionality [119], while a lack of cryoprotectants during frozen storage can remove biomolecules from the EV surface [120]. EV isolation techniques, such as shear stress‐controlled tangential flow filtration [121] and low‐flow‐rate size‐exclusion chromatography [122] are less likely to cause damage, making them ideal for assessing surface signaling.

Tracking the location of EVs through labelling with lipophilic dyes may aid is assessing the time scale of various forms of signaling (surface vs. internalized). However, such dyes can detach from EVs and bind to other structures, leading to misrepresented findings. Additionally, the dyes can self‐assemble into nanosized structures that are mistaken for EVs, which can lead to false positive results [123]. Beyond immediate readouts and fluorescent labeling of EVs, the use of broad‐spectrum endocytosis inhibitors, such as cytochalasin D, which acts through suppression of actin polymerization, can aid in assessing whether EV‐induced signaling in recipient cells is dependent on internalization [9]. However, it is possible that inhibition of actin polymerization also impacts cell interactions with EV surfaces and subsequent cell signaling. Another method that may shed light on the contributions of surface signaling vs. endocytosis is performing studies at low temperatures (< 10°C), which impedes the energy‐dependent process of endocytosis [124]. Similar to actin polymerization, low temperatures can also affect cell signaling pathways, making neither of these methods entirely reliable.

Beyond assessing the relative contributions of surface signaling vs. internalization, more sophisticated methods are required to demonstrate the ability of a single EV to transiently interact with multiple cells, impacting their function. Obtaining such experimental evidence would likely require single‐EV tracking methods combined with single‐cell response read‐outs. However, there is a scarcity of tools that can accurately assess EVs on the single‐particle level due to technical challenges related to nanoscale dimensions and heterogeneity within and between EV subpopulations. Uncovering the inter and intra‐EV distribution of surface biomolecules will be aided by tools, such as advancements in cryogenic transmission electron microscopy coupled with immunogold labelling and [125] direct stochastic optical reconstruction microscopy (dSTORM) [126]. There are also several complementary techniques that can provide insights into the spatiotemporal interactions between individual EVs and membrane‐embedded proteins (Table 2). A limitation of such approaches is that they often use synthetic phospholipid membranes that in many ways fail to mimic a physiological cell membrane. In cases where live cells are used, an artificial physical environment (for example, cells immobilized on a stiff two‐dimensional surface) and a non‐physiologically representative extracellular fluid, could also perturb EV signaling [127]. Combining reporter‐cell systems with fluorescent and biophysical single‐particle tracking techniques holds promise for obtaining future evidence of sequential bind‐and‐leave signaling.

**TABLE 2:** Examples of techniques with potential to assess EV surface signaling.

Technique	Description	Advantages	Disadvantages	Refs.
dSTORM	Single‐molecule localization microscopy technique with a spatial resolution of 20 nm or better Achieves super‐resolution imaging by exploiting the stochastic blinking of fluorophores Can be used to visualize EVs through fluorescent antibodies, such as those targeting CD9 and CD81	Surpasses the diffraction limit of conventional fluorescence microscopy (∼250 nm lateral resolution) Single‐particle and spatial resolution (distinguish individual EVs from aggregates and visualize membrane microdomains)	Low throughput High‐cost instrumentation Requires advanced software and technical expertise Partially destructive Dyes may suffer from photobleaching and variable blinking kinetics, leading to localization errors and misinterpretation of the molecular organization of EV domains EVs can be susceptible to labeling inefficiencies due to small size and heterogeneous nature	[126, 128]
Extracellular vesicle‐Target cell Interaction Detection through SorTagging (ETIDS)	Reporter‐cell system EVs are produced from cells expressing a ligand of interest fused to an enzyme (sortase A) Another set of cells express a receptor of interest fused to a tag that accepts the enzymatic transfer of the biotin‐labelled substrate Cells only become biotin‐labelled if the EV ligand is in physical contact with the cell receptor	Standard cell biology tools (for example, flow cytometry) can be used for detection High throughput Non‐destructive Performed in physiologically relevant settings (live cells) Does not require immobilization of binding partners	Requires genetic engineering of fusion constructs (not applicable to native receptors/ligands without modification) Lacks single‐particle and spatial resolution Dependent on efficient tagging and enzyme accessibility	[32]
Total internal reflection fluorescence microscopy (TIRFM)	Selectively excites fluorophores within ∼100‐200 nm of a glass‐membrane interface by generating an evanescent field Used to assess interactions between fluorescently labelled EVs and protein ligands embedded in a lipid bilayer Only fluorophores close to the membrane (for example, bound EVs) emit fluorescence	Single‐EVs can be visualized Non‐destructive Minimal background fluorescence from cytoplasmic regions	High‐cost instrumentation Relies on fluorescent tags on EVs Lacks nanoscale separation Requires immobilisation of one binding partner	[38]
ExoScreen	Uses donor and acceptor beads conjugated to antibodies targeting EV surface proteins Generates a fluorescent signal when both antibodies bind epitopes within 200 nm, indicating close molecular proximity Has potential to be adapted for probing EV‐cell surface interactions by pairing antibodies against EV markers and cell‐surface receptors, with signal indicating physical docking	High throughput Standard laboratory tools (for example, fluorescent plate reader) can be used for detection Performed in physiologically relevant settings Does not require immobilization of binding partners Non‐destructive	Lacks single‐particle and spatial resolution EVs can be susceptible to labeling inefficiencies due to small size and heterogeneous nature Has not yet been used to assess EV‐cell interactions	[129]
Lateral flow immunoassay (LFIA)	Uses capillary flow to transport EV samples across a membrane with immobilized capture antibodies targeting EV surface proteins Generates a visible signal when EVs bind to capture antibodies and are detected by labeled secondary antibodies (gold nanoparticles) Has potential to be adapted for probing EV binding to cell receptors/ligands	High throughput Does not require sophisticated equipment Non‐destructive	Limited to simplified model systems (not live cells) Lacks single‐particle and spatial resolution Requires immobilization of one binding partner	[130]
Microfluidic immunoaffinity with nanoshearing (can be combined with surface‐enhanced Raman scattering/SERS)	Uses alternating current‐induced electrohydrodynamic forces to generate nanoscale shear flow within a few nanometers of an antibody‐functionalized electrode surface Enhances specific EV‐antibody interactions by increasing collision frequency while removing weakly bound, nonspecific species Enables multiplexed, on‐chip EV detection with colorimetric readout Readout can also be performed with SERS (EVs labeled with gold nanoparticles) Has potential to be adapted for probing EV binding to cell receptors/ligands	High throughput High‐sensitivity detection of surface‐mediated interactions in complex biofluids Non‐destructive	Limited to simplified model systems (not live cells) Requires immobilization of one binding partner	[131, 132]
Atomic force microscopy (AFM)	Uses a sharp cantilever tip that physically scans across a surface to map topography, stiffness, and interaction forces at nanometre resolution Functionalised AFM tips can be coated with receptors or ligands to measure unbinding forces Can produce adhesion maps of EV binding and assess binding strength	Label‐free Single‐particle and spatial resolution Can be combined with dSTORM to enable multi‐color labeling and colocalization of structural and molecular components of EVs	High‐cost instrumentation Requires advanced software and technical expertise Low throughput Tip pressure can deform soft biological samples Requires immobilisation of one binding partner	[133, 134, 135]
Small‐angle X‐ray and neutron scattering (SAXS/SANS)	SAXS measures the scattering of X‐rays as they pass through a sample; with scattering occurring due to variations in electron density SANS measures the scattering of neutrons, which interact with the nuclei of atoms Assesses nanoscale structural changes in the membrane architecture, indicative of modifications in membrane bilayer structure, such as lipid reorganization, bilayer thickness alterations, and domain formation induced by EV binding and fusion	Label‐free Non‐destructive Does not require immobilisation of of binding partners	High‐cost instrumentation Requires advanced software and technical expertise Lacks single‐particle resolution Requires immobilisation of one binding partner	[136]
Neutron reflectometry	Provides detailed information about the thickness, density, and roughness of layers by measuring the reflectivity of a neutron beam as it strikes a sample at a shallow angle Enables identification of preferred membrane docking sites for EVs	Label‐free Non‐destructive	High‐cost instrumentation Requires advanced software and technical expertise Low throughput Lacks single‐particle resolution Limited to simplified model systems (not live cells) Requires specialized neutron facilities	[136]
Quartz crystal microbalance (QCM)	Detects frequency shifts in a quartz crystal as mass accumulates on its surface Interaction with a supported lipid bilayer (model cell membrane) causes measurable frequency changes according to Sauerbrey's equation Monitoring dissipation reveals the bilayer's viscoelastic properties Enables quantification of EV binding events and assessment of membrane mechanical changes	Label‐free Non‐destructive High sensitivity for adsorption kinetics	Lacks single‐particle and spatial resolution Requires immobilisation of one binding partner Susceptible to non‐specific binding and buffer artefacts	[137, 138, 139]
Surface plasmon resonance (SPR)	Uses excitation of surface plasmons‐electron oscillations at the metal‐dielectric interface to detect molecular binding events Binding of molecules to the metal sensor alters the reflection angle and reduces light intensity The change in light intensity is proportional to bound mass, allowing quantitative analysis of binding affinity, kinetics, and membrane responses to EV attachment	Label‐free Non‐destructive High sensitivity	High‐cost instrumentation Lacks single‐particle and spatial resolution Requires immobilisation of one binding partner	[140, 141]
Holotomography	Coherent light sources, such as lasers, illuminate the sample and capture the resultant interference patterns Holographic images from multiple angles are recorded and reconstructed computationally to form a nanoscale three‐dimensional image	Label‐free Non‐destructive Does not require immobilisation of of binding partners Can be combined with fluorescent imaging for detailed and real‐time three‐dimensional visualization of stained EV interactions with cells	High‐cost instrumentation Requires advanced software and technical expertise The resolution limit of holotomography makes it unsuitable for small EVs (< 200 nm)	[39]

## 6 Outlook

Many unresolved questions remain regarding the modes and effectiveness of EV signaling. EVs have been demonstrated to signal through both cellular internalization [15, 16, 17] and surface signaling [30, 36], however, the relative contributions of each and to what extent individual EVs signal through multiple modes remains unknown. It is likely that single EVs use several signaling types; initially through a bind‐and‐leave mechanism, followed by a bind‐and‐stay and/or bind‐and internalize mechanism. Similarly, it remains unknown whether different EV biogenesis pathways are more closely associated with specific modes of signaling, and which EV characteristics determine signaling mechanisms. It is tempting to speculate that EVs inherit signaling features associated with their origin. For example, ectosomes, which form by outward budding of the plasma membrane, may retain surface‐signaling domains present on the originating cell membrane. In contrast, exosomes, which come from the endocytic pathway, may have an increased tendency for uptake‐dependent signaling, consistent with their intracellular trafficking origin. Beyond biogenesis, the molecular and physical attributes of EV subpopulations are known to impact signaling [142], indicating multifactorial complexity. An area of future research in EV signaling is the identification of biomarkers that predict the propensity of EVs to signal via surface interactions vs. internalization.

**FIGURE 4:**
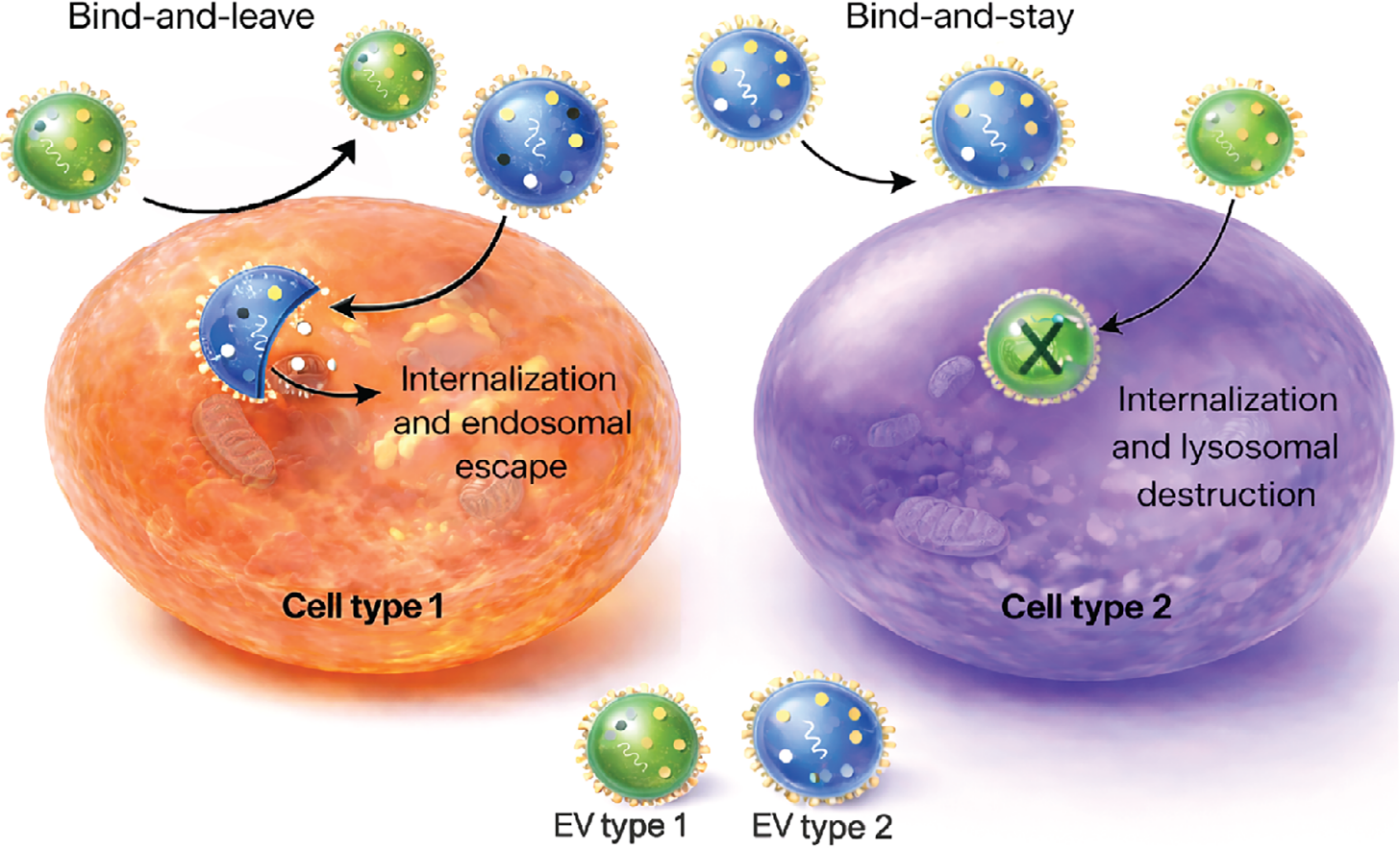
EV‐recipient cell pairings dictate the signaling type. Emerging evidence suggests that pairings between EVs and recipient cells determines whether signaling occurs and which mode is used. EV types 1 and 2 denote populations with distinct surface compositions.

Signaling modes are also likely to be highly context‐dependent differing based on the recipient cell and tissue microenvironment. For example, in cells or tissues with high phagocytic activity, EV internalization may be the most prominent mechanism of EV‐mediated effects. A study showed that EVs isolated from microglia were internalized by other microglia (phagocytic cells), while these EVs remained on the surface of astrocytes [143]. Similarly, leukemia‐derived EVs were internalized by phagocytic cells, but primarily attached to and remained on the surface of non‐phagocytic cells [144]. Accordingly, the specific EV‐recipient cell pairing is likely to dictate the mode and biological effects of signaling (Figure 4), adding a further level of complexity. It should also be noted that the specificity of EVs toward cell types is likely overstated in the current literature, and in many cases may be driven by non‐specific interactions.

It is important to emphasize the distinction between efficiency and effectiveness in EV signaling. Surface signaling (bind‐and‐leave) is an efficient signaling mode in terms of one EV being able to affect multiple cells, while internalization is inefficient due to high levels of lysosomal destruction [8, 11, 12] and a “one‐vesicle‐one‐cell” mechanism. It remains unclear which EV signaling mode is the most effective, and this is likely to be context‐dependent. A potential mechanism to compensate for the inefficiency of internalization‐based signaling is signal amplification through the release of secondary EVs in response to uptake of primary EVs [145, 146]. For example, the effects of suppressor T cell‐derived EVs was dependent on macrophage internalization and release of secondary EVs that suppressed effector T cells [147, 148]. This concept builds on earlier speculation that macrophages may function as a central sorting facility with the capacity to either destroy or re‐package EV messages [149], with secondary EVs enabling signal amplification.

The nanoscale dimensions and heterogeneity within and between EV subpopulations make it technically challenging to decouple different modes of signaling and the contributions of each to biological outcomes, although several emerging techniques are currently being developed. It is important to note that model systems that are used for assessing interactions between EVs and cells are merely approximations of real systems. Consequently, findings evaluating EV efficiency and effectiveness in synthetic membranes may fail to correlate with those in cell membranes, while in vitro experimental data may fall short of predicting in vivo outcomes. Future technical advances coupling EV‐cell surface binding kinetics and internalization with functional in vitro and in vivo studies are likely to determine the effectiveness of signaling through ‘bind‐and‐leave’ vs. cellular uptake mechanisms. Additionally, new techniques may uncover EV membrane domains with multiple biomolecules that act as potent signaling hubs.

It remains unclear which signaling modes are predominantly responsible for the therapeutic effects of EVs, and some therapeutic mechanisms may operate independently of direct cellular interactions, such as EV‐mediated degradation of extracellular adenosine triphosphate [95] and destabilization of complement protein complexes [150] as anti‐inflammatory mechanisms. Dosing and optimal numbers of EVs per target cell are likely to be highly dependent on signaling modes and remain key considerations for therapeutic EVs. Recently, it was shown that signaling through EV internalization is dose‐dependent, with higher doses, paradoxically, eliciting increased lysosomal activity and diminished effects [151]. In general, dose‐dependent effects may be challenging to interpret, as one mode of EV signaling may be enhanced with higher doses (surface signaling), while other modes (internalization) may be suppressed beyond a certain dose threshold.

To fully realize the therapeutic potential of EVs, it may be necessary to understand, exploit, and engineer all modes of signaling. Complete understanding of EV therapeutic signaling is dependent on uncovering the role of EV heterogeneity, which is usually considered a barrier for therapeutic development. EV preparations contain various subpopulations, each of which is likely to trigger distinct signaling outputs. However, EV heterogeneity may be key to multifunctionality [152, 153], which is considered a key factor underlying the potential of EVs to outperform conventional medicines. Multifunctionality is key to effectively impacting biological processes, which often require simultaneous activation and/or inhibition of multiple pathways. Therefore, finding a balance between minimizing heterogeneity for regulatory consistency and leveraging heterogeneity for therapeutic signaling is key.

The use of multiple innovative techniques and systematic approaches to decoupling EV signaling hold promise for uncovering the roles of signaling modalities in driving specific outcomes, which will expand basic knowledge and support therapeutic development. Going forward, we recommend that researchers remain mindful of guidelines from the International Society of Extracellular Vesicles [154] for standard EV procedures (such as EV authentication), while also pursuing innovative approaches beyond current norms to generate the evidence that will shape future guidelines.

## Funding

Partial funding For this work was provided by the University of Queensland, Australia (J.W.), the National Breast Cancer Foundation, Australia, under award number 2023/IIRS0063 (J.W.), the National Heart Foundation of Australia under award number 108500‐2024_FLF (J.W.), the Medical Research Future Fund, Australia, under award number MRF2019485 (J.W.), the Leo Foundation, Denmark, under award number LF‐OC‐25‐002046 (W.C.), the Wenner‐Gren Foundation, USA, under award number GFO2023‐0008 (W.C.), and National Health and Medical Research Council, Australia, under award number 2037119 (W.C.). The content is solely the authors’ responsibility and does not necessarily represent the official views of the organizations and funding agencies.

## Conflicts of Interest

J.W. is currently or has previously served as a board member or scientific advisor for biomedical companies, including Omnidermal, Genomill, and Pharmatest Services. The main focus of these companies is not on extracellular vesicles or nanomedicine. Ionis Pharmaceuticals, Sartorius, and Sanofi have sponsored or are sponsoring extracellular vesicle or nanomedicine research in the Wolfram Laboratory.

## Data Availability

The authors have nothing to report.
